# Maternal total cell-free DNA in preeclampsia and fetal growth restriction: Evidence of differences in maternal response to abnormal implantation

**DOI:** 10.1371/journal.pone.0200360

**Published:** 2018-07-12

**Authors:** Tal Rafaeli-Yehudai, Majdi Imterat, Amos Douvdevani, Dan Tirosh, Neta Benshalom-Tirosh, Salvatore Andrea Mastrolia, Ruthy Beer-Weisel, Vered Klaitman, Reut Riff, Shirley Greenbaum, Alex Alioshin, Gal Rodavsky Hanegbi, Giuseppe Loverro, Mariana Rita Catalano, Offer Erez

**Affiliations:** 1 Department of Obstetrics and Gynecology, Soroka University Medical Center, School of Medicine, Ben Gurion University of the Negev, Beer Sheva, Israel; 2 Department of Clinical Biochemistry and Pharmacology, Soroka University Medical Center, School of Medicine, Ben Gurion University of the Negev, Beer Sheva, Israel; 3 Department of Obstetrics and Gynecology, Azienda Ospedaliera Universitaria Policlinico di Bari, School of Medicine, University of Bari "Aldo Moro", Bari, Italy; 4 Department of Maternal Fetal Medicine, Fondazione MBBM, San Gerardo Hospital, School of Medicine, University of Milano Bicocca, Monza, Italy; 5 Maternity Department D, Obstetrical Day Care Unit, Soroka University Medical Center, School of Medicine, Ben Gurion University of the Negev, Beer Sheva, Israel; Virgen Macarena University Hospital, School of Medicine, University of Seville, SPAIN

## Abstract

**Objectives:**

Preeclampsia and fetal growth restriction are obstetrical syndromes associated with abnormal placental implantation and changes in the activation status of maternal leukocytes. This study is aimed to determine by a simple, rapid fluorescent assay the changes in maternal serum total cell-free DNA (t-cfDNA) concentrations in women with preeclampsia and those with fetal growth restriction (FGR).

**Study design:**

A cross-sectional study was conducted measuring maternal serum t-cfDNA concentrations. Women were classified into the following groups: 1) patients with preeclampsia (n = 21); 2) FGR-estimated fetal weight below the 10^th^percentile (n = 28); and 3) normal pregnancy (n = 39). Serum samples were directly assayed for t-cfDNA using a rapid fluorescent SYBR Gold assay. Elevated maternal serum t-cfDNA concentrations were defined as a cutoff>850ng/ml. Nonparametric statistics were used for analysis.

**Results:**

Women with preeclampsia had a higher median maternal serum concentration (802 ng/ml, 400–2272 ng/ml) than women with a normal pregnancy (499 ng/ml, 0–1892 ng/ml, p = 0.004) and those with FGR (484 ng/ml, 72–2187 ng/ml, p = 0.012). Moreover, even patients with FGR <5^th^ percentile and abnormal Doppler had a lower median maternal serum t-cfDNA than those with preeclampsia (median 487 ng/ml, 144–1971 ng/ml, p = 0.022). The median concentration of t-cfDNA did not differ between women with a normal pregnancy and those with FGR (p = 0.54), as well as those with fetuses <5th percentile and abnormal Doppler (p = 0.7). Women with preeclampsia had a higher proportion of elevated t-cfDNA than those with a normal pregnancy (p = 0.015) and patients with FGR (p = 0.025).

**Conclusions:**

Preeclampsia is associated with higher maternal serum t-cfDNA concentration than normal pregnancy or FGR. This observation may reflect an increased systemic activation of the maternal inflammation, rather than placental; this assumption is supported by the fact that we did not observe a significant change in the maternal serum t-cfDNA in patients with placental-mediated FGR.

## Introduction

Circulating cell-free nucleic acids in plasma and serum are considered novel biomarkers with promising clinical applications in different medical conditions [1–9]. These biomarkers were reported in various aspects of obstetrics (especially in prenatal diagnosis) and in adverse pregnancy outcomes [10–13]. In serum samples, the vast amount of total cf-DNA is derived from the demise of maternal leucocytes [14]. This is why there is such a huge difference in the amount of this material with matching plasma samples. Placental or fetal cf-DNA (cfp-DNA) makes up for a small fraction (≈5%) of the total cf-DNA pool in maternal plasma, and is even lower in serum samples, as it is diluted by the significant increase in maternal leucocyte derived material [15]. The cfp-DNA can be detected in maternal circulation as early as the fifth or sixth weeks of gestation [16]. Its concentrations increase steadily with advancing gestational age. Maternal leucocyte, mainly neutrophils are the source of the elevated concentrations of cfDNA in the serum reflecting the enhanced capability of these cells to form Neutrophil Extracellular Traps (NETs) [14]. The small fragments of cfp-DNA predominantly originate from trophoblast cells [14, 17, 18]; nevertheless, the quantification of circulating cfp-DNA in maternal circulation requires the utilization of complex methods for DNA extraction, and real-time-polymerase chain reaction (rt-PCR). Our group previously reported [19–22] a rapid direct fluorescent assay for total cf-DNA concentration in biological fluids that uses the SYBR® Gold dye. Our test does not require a prior processing of the samples and therefore is a potential "point of care" assay.

Preeclampsia and fetal growth restriction (FGR) represent two of the “Great Obstetrical Syndromes”[23], constituting one of the leading causes of maternal and fetal/neonatal morbidity and mortality worldwide [24, 25]. These two conditions are the clinical end point of many underlying mechanisms (different as well as similar) and are frequently associated with one another [23]. The similar underlying mechanisms leading to FGR and preeclampsia include: 1) abnormal placentation, manifested as failure of transformation of the spiral arteries and shallow trophoblast invasion[26–28]; 2) an imbalance between angiogenic and anti-angiogenic factors in maternal blood[29–39]; 3) chronic utero-placental ischemia [40–42]; 4) increased trophoblast apoptosis/necrosis [43]; and 5) an enhanced maternal systemic inflammatory response [44–46]. However, In spite of these similarities in the underlying mechanisms, the two obstetrical syndromes differ in their clinical manifestation. [47].

Conditions that affect the placenta can directly impact its concentrations of cell-free fetal DNA in the maternal circulation, as its release is closely related to placental morphogenesis [48]. Preeclampsia is associated with elevated concentrations of cell-free fetal DNA in maternal circulation [49–55]. In pregnancies complicated with FGR, the cell-free fetal DNA concentrations were either normal or increased (yet to a lesser extent than that observed in early-onset preeclampsia) [56–59].

The aim of our study was to determine the changes in maternal serum total cell-free DNA (t-cfDNA) in women with preeclampsia and in women with FGR by using the fluorochrome SYBR® Gold assay.

## Materials and methods

### Study groups and inclusion criteria

A prospective cross-sectional study was conducted at the Soroka University Medical Center, a tertiary medical center in the southern region of Israel, which serves the entire obstetrical population of the region. Serum samples for total cf-DNA were collected from patients in the following groups: 1) women with normal pregnancies (n = 39); 2) patients with preeclampsia (n = 21); 3) patients with isolated FGR (without PE)(n = 28). Patients with multiple pregnancies or fetuses with congenital and/or chromosomal anomalies were excluded. All women provided a signed, informed consent prior to the collection of maternal blood. The Institutional Review Board of the Soroka University Medical Center approved the study.

#### Data collection

Information regarding the pregnancy and delivery outcomes was collected from the birth files and medical records of the patients. Raw data is presented in S1 File.

### Clinical definitions

*Normal pregnancy* was defined as women who delivered at term (37–42 weeks) a singleton neonate with a 5-minute Apgar score ≥7 and a birthweight between the 10^th^ and 90^th^ percentiles, and had no medical or obstetrical complications during pregnancy.

Preeclampsia was defined in the presence of new-onset hypertension in the second half of pregnancy (systolic blood pressure ≥140 mmHg or diastolic blood pressure ≥90 mmHg on at least two occasions, 4 hours to 1 week apart) and proteinuria (≥300 mg in a 24-hour urine collection or protein/creatinine ratio of at least 0.3 [each measured as mg/dl] or dipstick measurement ≥ 1) [60].

For the purpose of fetal growth evaluation, gestational age dating was primarily defined by last menstrual period (LMP) and correlated, when possible, with 1st trimester crown-rump length (CRL). If there was a difference of ≥7 days between gestational age by LMP and 1st trimester CRL gestational dating was set according to CRL measurements [61]. *Fetal growth restriction (FGR*) was defined as estimated fetal weight below the 10^th^ percentile with or without abnormal umbilical, uterine, and middle cerebral artery Doppler velocimetry [62].

### Sample collection and cell free DNA rapid direct fluorescent assay

Blood samples were collected using BD Vacutainer® gel tubes (Becton- Dickinson, Plymouth, UK) and samples were stored at 4°C for up to 24 hours before centrifugation. Serum was separated from the cellular fraction and frozen at -20°C until assayed. Total cf-DNA was detected directly in sera, according to our method [19]. Briefly, 20 μL of sera or DNA standard solutions were applied in duplicate to black 96-well plates (Greiner Bio-One; Frickenhausen, Germany). 80 μL of diluted SYBR® Gold was added to each well (final dilution 1:10.000), and fluorescence was measured with a 96-well fluorimeter at an emission wavelength of 535 nm and an excitation wavelength of 485 nm. Concentrations of unknown samples were calculated from a standard curve by extrapolation in a linear regression model. As described previously our assay correlates with the conventional quantitative PCR assay of ß-globin (R2 = 0.9987, p<0.001) [19].

### Statistical analysis

Statistical analysis was performed with the SPSS package, version 20 (SPSS, Inc.; Chicago, IL). Statistical significance of the categorical variables was tested using the *x*^2^ or Fisher’s exact test, as appropriate. Since data was not normally distributed, we used non-parametric statistics for the analysis of continuous variables, including Man–Whitney and Kruskal Wallis tests. Cox proportional hazard regression analysis was used to calculate the gestational age adjusted hazard ratio for the occurrence of preeclampsia according to the concentrations of total free cell DNA. A P value< 0.05 was considered statistically significant.

High total cell-free DNA concentrations were defined as a serum concentration above 850ng/mL, which represents a value of two standard deviations above the mean, previously reported in healthy adults using the same assay [22, 63, 64].

## Results

Of the total 88 pregnancies that met the inclusion criteria, serum samples for total cf-DNA were collected from 21 patients with preeclampsia, 28 patients with FGR (14 of them ≤5th percentile), and 39 women with normal pregnancies.

Table 1 displays the demographic and clinical characteristics of the study groups. Patients with FGR had lower median gestational age at sample collection (p = 0.005), than women in the other study groups. Patients with preeclampsia had significantly higher rates of history of preeclampsia than women with either a normal pregnancy or FGR (p = <0.001). No significant differences were found among the study groups regarding maternal age, maternal BMI, ethnicity, gravidity, parity, smoking, diabetes, and history of SGA neonates, or the use of assisted reproductive technologies.

**Table 1:** Demographic and clinical characteristics of the study population.

Variable	Control(n = 39)	Preeclampsia (n = 21)	FGR(n = 28)	p-value
**Maternal Age**	30.3 ± 5.2	28.9 ± 5.6	27.8 ± 8	0.143
**Ethnicity Bedouine origin**	27.5 (11)	42.9 (9)	42.9 (12)	0.315
**BMI**	25.5 ± 5.6	29.1 ± 7.5	26.7 ± 5.3	0.172
**Gravidity**	3 (1;15)	1 (1;9)	2 (1;7)	0.175
**Parity**	1.5 (0;9)	0(0;7)	0 (0;5)	0.131
**Gestational Age at sample collection (weeks)**	35.4 ± 4.6	34.9 ± 4	32 ± 4.7	0.005
**Infertility treatments**	5 (2)	14.3 (3)	10.7 (3)	0.656
**History of preeclampsia**	0 (0)	19 (4)	0 (0)	<0.001
**History of IUGR**	2.5 (1)	5 (1)	7.1 (2)	0.692
**Diabetes**	7.5 (3)	4.8 (1)	10.7 (3)	0.858
**Smoking**	10 (4)	0 (0)	21.4 (6)	0.06

Data are presented as median (min;max), mean± SD, percentage (number). IUGR, intrauterine growth restriction.

Table 2 displays the perinatal outcome among the study population. Patients with FGR had lower median gestational age at delivery (p = <0.001) and lower median birthweight (p = <0.001) than women with normal pregnancies and women with preeclampsia. There was no significant difference in the low Apgar scores at 1 and 5 minutes among the groups.

**Table 2:** Perinatal outcomes among the study groups.

Variable	Control	Preeclampsia	FGR	p-value
**Gestational age at delivery (weeks)**	39±1.5	35.3±3.7	34.8±4.2	<0.001
**Neonatal Birthweight (gr)**	3189.8±450.1	2232.6±753.1	1652.7±727.6	<0.001
**Apgar score <5 at 1'**	2.6(1)	9.5(2)	16.7(4)	0.172
**Apgar score <7 at 5'**	0(0)	9.5(2)	8.3(2)	0.221

Data are presented as median (min;max), mean± SD, percentage (number). IUGR, intrauterine growth restriction.

### Changes in the median maternal serum concentrations of total cf-DNA in the different study groups

**Fig 1:**
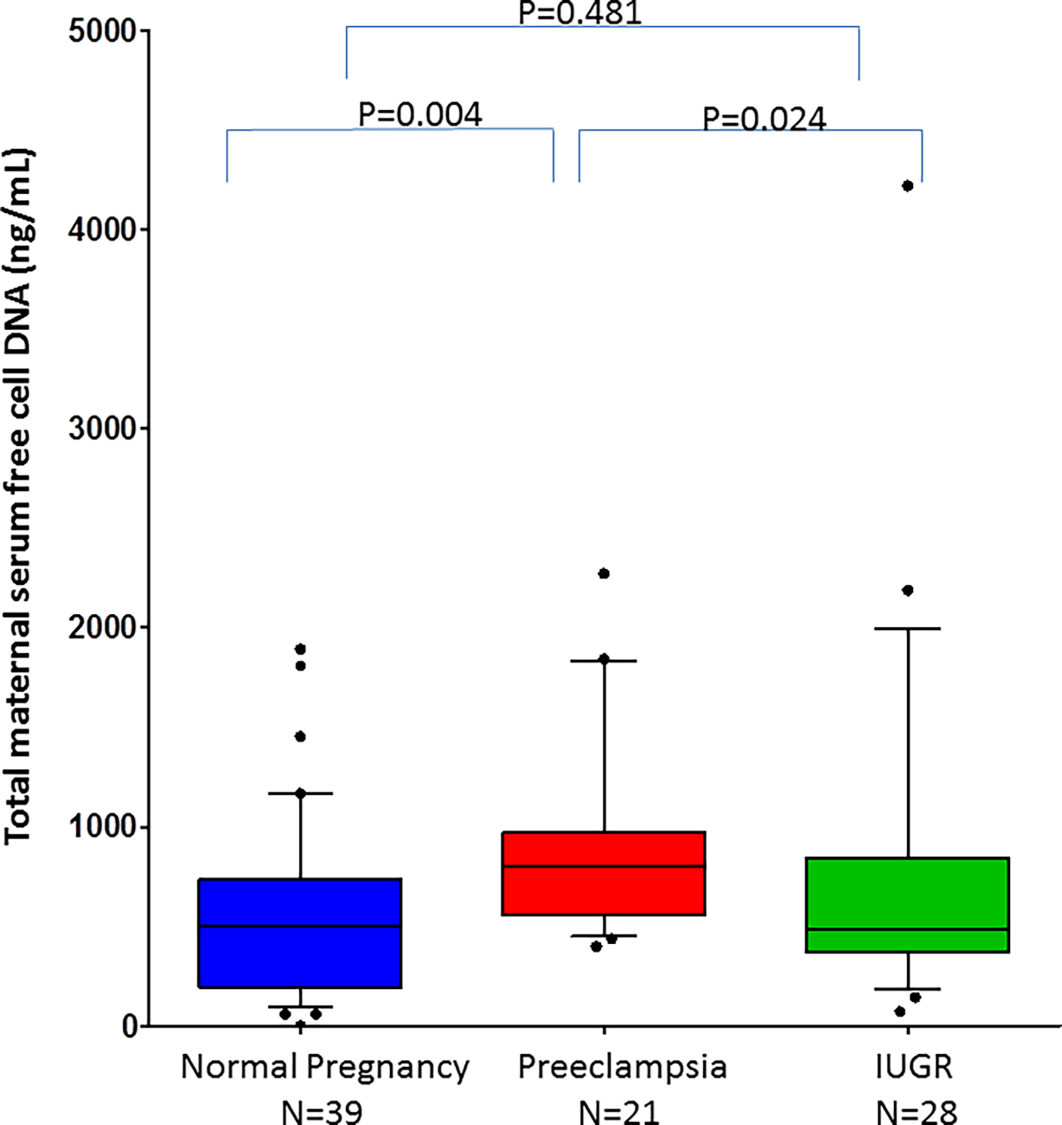
Median maternal serum concentration of study groups.

Maternal serum total cf-DNA concentrations differed significantly among the study groups (Kruskal-Wallis Test p = 0.006). The median maternal serum total cf-DNA concentrations were higher in patients with preeclampsia than in women with normal pregnancies (median:802 ng/ml; range 400–2272 vs.median:499 ng/ml;range 0–1892; p = 0.004, respectively) as well as from patients with FGR (median: 484 ng/ml; range 72–4219; p = 0.024) (Fig 1).

**Fig 2:**
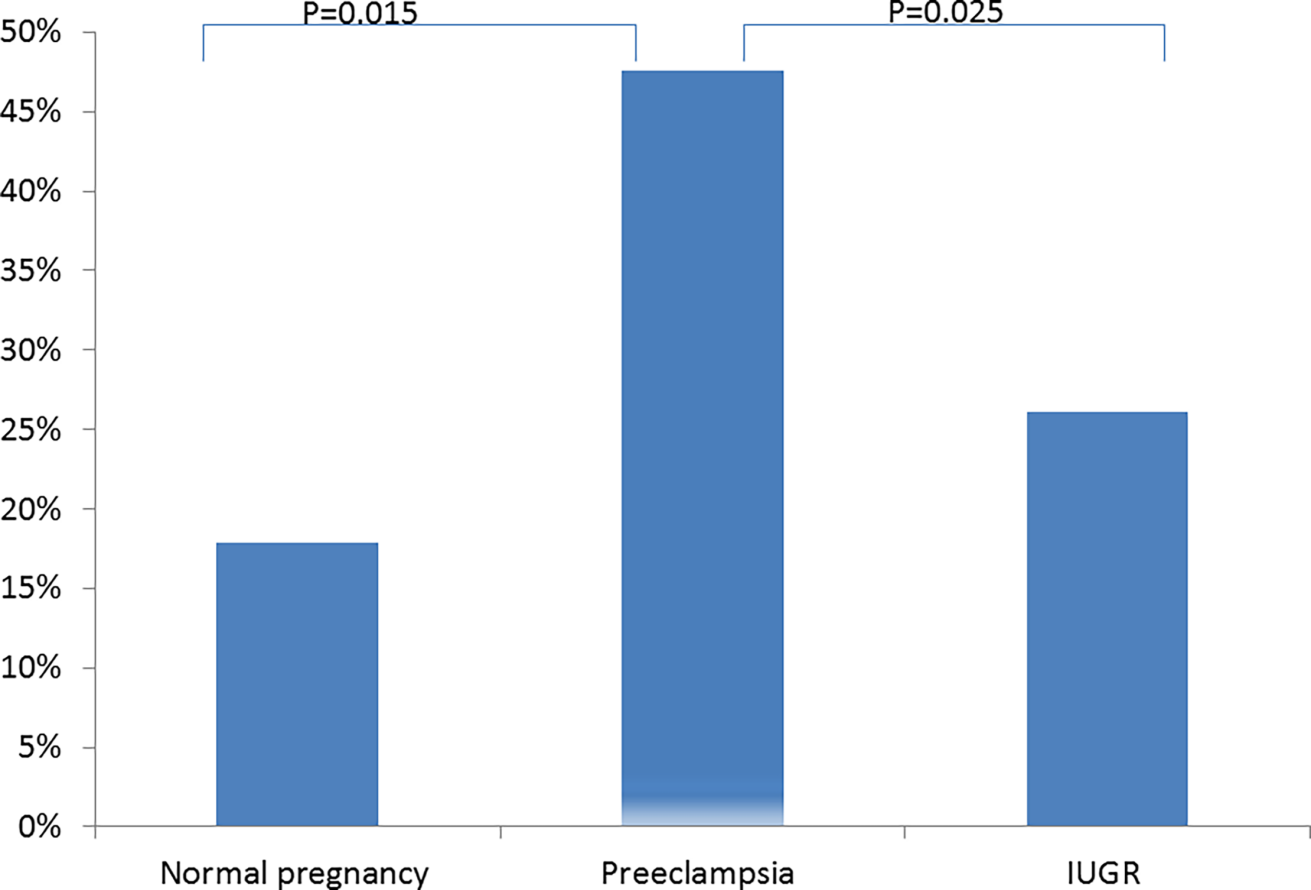
Elevated rate of maternal serum concentration of study groups.

The rate of elevated total cf-DNA concentration was higher in women with preeclampsia than in those with both normal pregnancy (p = 0.015) and FGR (p = 0.02.5) (Fig 2).

**Fig 3:**
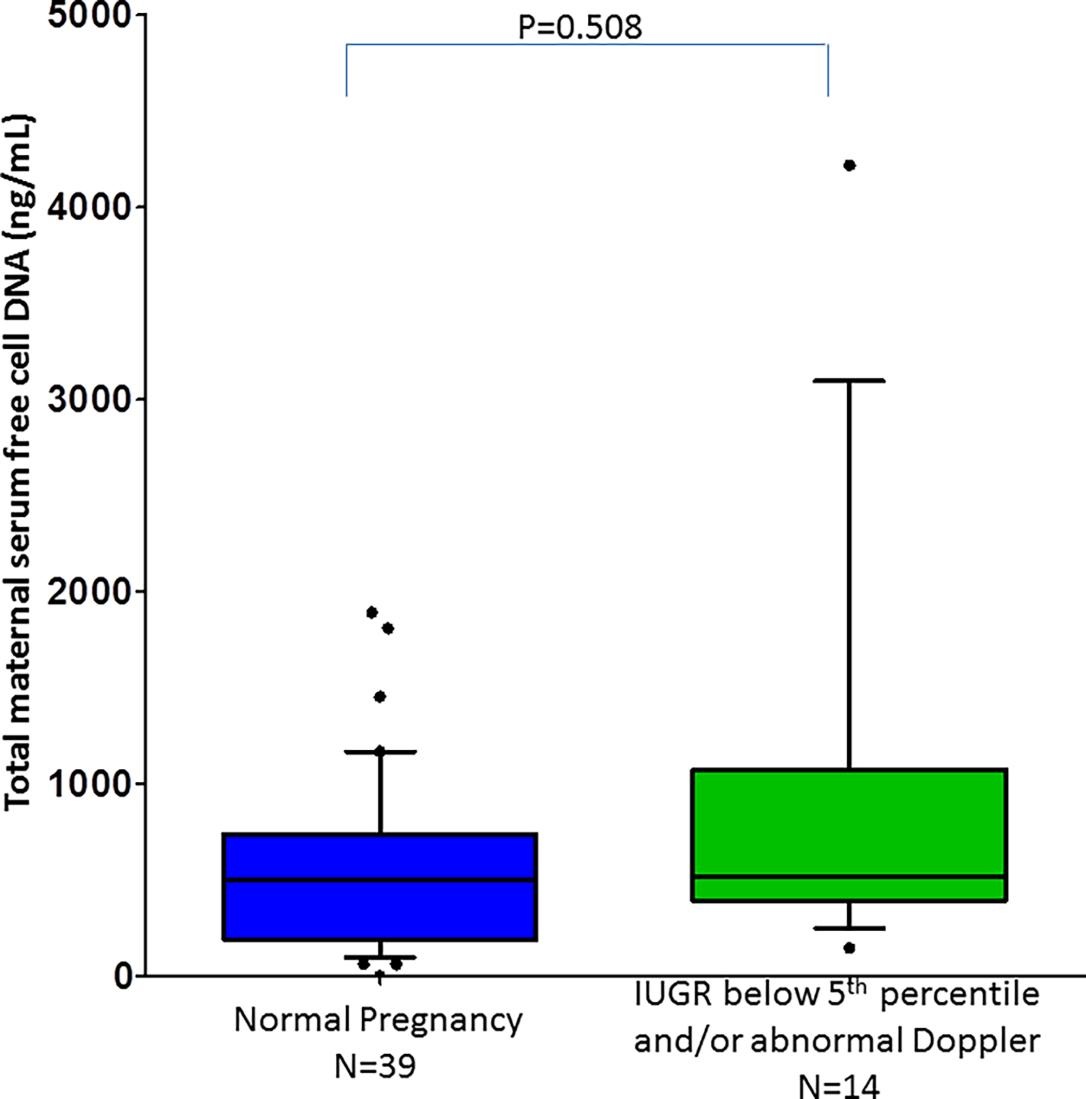
Median maternal serum concentration of normal pregnancies and pregnancies with IUGR below 5^th^ percentile.

Moreover, the comparison between patients with preeclampsia and those with FGR ≤5th percentile, who had abnormal Doppler, yielded similar results (487 ng/ml, 144–1971 ng/ml, p = 0.022). The median concentration of total cf-DNA did not differ between women in the normal pregnancy group and those with FGR (p = 0.481), even when only those with fetuses ≤5th percentile and abnormal Doppler were included (p = 0.508) (Fig 3).

We used a Cox regression analysis to calculate the risk associated with total free cell DNA to developed preeclampsia according to gestational age after adjustment for confounding factors such maternal age and BMI. Increased maternal serum total cf-DNA concentrations (ng/ml) were independently associated with preeclampsia (Hazard ratio (HR) for preeclampsia of 1.002 (95% confidence interval 1.0003–1.003) while maternal BMI and age did not reach statistical significance (date not shown). In addition elevated cf-DNA was associated with an HR for preeclampsia of 12.36 (95%CI 2.53–60.41).

## Discussion

### Principal findings of the study

We found that women with preeclampsia have a higher median maternal serum total cf-DNA concentration than those of patients with normal pregnancy or those with pregnancies complicated with FGR, even if there were signs for placental origin of the FGR.

### What are the changes in circulating cf-DNA in preeclampsia and FGR?

The observation that the total maternal serum cf-DNA is higher in women with preeclampsia than in women with a normal pregnancy or those who had an FGR neonate is novel. The elevation in maternal as well as fetal cf-DNA concentration in the maternal plasma and serum of patients with preeclampsia was previously reported [44, 49–52, 54, 55, 65–70].

In addition, several studies examined the relationship between FGR and cf-DNA levels. Sekizawa, et al [56] examined the concentration of total and cf-DNA in 9 pregnancies complicated by FGR and in 9 pregnancies complicated by preeclampsia; they found that the concentration of cf-DNA in FGR pregnancies were similar to those in women with normal pregnancies. In contrast to our study, however, the authors could not demonstrate a significant difference in total concentrations of cf-DNA between patients with preeclampsia and those with FGR. Since Sekizawa et al were using plasma and not serum, it is not surprising that their data do not match those of the current study, where serum was used.

### Placental contribution to total cf-DNA

The human placenta is a dynamic organ with constant turnover of villous trophoblast. The turnover of villous trophoblast results in its final stage, in extrusion of apoptotic material (including cf-DNA) into the maternal circulation. In normal pregnancies, at term, grams of placental material are shed daily into the maternal circulation without causing inflammatory response [71]. As aforementioned, the release of cf-DNA is tied strongly to placenta morphogenesis. In cases of failure of the key mechanisms involved in placental development (proliferation, migration, invasion, and differentiation), such as in the case of preeclampsia, there are changes in placental cell composition, production of regulatory molecules, and cell turn-over, all of which directly impact the types and levels of placental inflammatory syncytiotrophoblast debris that enters maternal circulation [48, 71]. Furthermore, the report by Goswami et al. [72], increased concentration of syncytiotrophoblast microparticles was specific to women with preeclampsia, and not to those with normotensive FGR. This is in accord with our findings also suggesting difference in the pathophysiology of these two syndromes.

Apoptosis appears to be the main mechanism controlling release of cf-DNA from the placenta. As a final event during the apoptosis cascade, old and late apoptotic nuclei are packed and released from syncytiotrophoblast in the form of exosomes to the maternal circulation [71, 73]. In addition to apoptotic mechanisms, accidental breakage or necrosis may also lead to the release of cf-DNA [65]; indeed, preeclampsia is associated with an increased placental apoptosis that leads to the apoptotic shedding and enhanced release of non-apoptotic molecules [71]. Our findings question the concept that the elevated total maternal cf-DNA observed in patients with preeclampsia is generated from the placenta. This doubt increased when we surveyed the results of the FGR groups: we could not demonstrate that patients, who had an FGR <5^th^ percentile and abnormal Doppler (representative of placental origin), had an elevation in the median total circulating cf-DNA. This finding is in contrast to the report of Al Nakib et al [57], who demonstrated an increase in the total cf-DNA concentration in 19 pregnancies complicated by FGR due to placental origins. A possible explanation of the differences between the two studies is the method of measurement and sample size in each study group. Collectively we can summarize that even in cases of severe preeclampsia or placental FGR with evidence of substantial trophoblast apoptosis the overall contribution of fetal/placental DNA to the total circulating DNA in the maternal serum is minor.

### Systemic maternal inflammation and changes in cf-DNA concentrations

In both obstetrical syndromes (preeclampsia and FGR), there is an enhanced maternal systemic inflammatory response; however, the magnitude and characteristics of this response differed between the two entities [44, 45, 74–76]. Oggé et al. [44] reported that patients with preeclampsia had a lower median mean channel brightness of CD62L on granulocytes as well as higher median basal radical oxidative species and oxidative bursts on monocytes than in patients with FGR. They concluded, in pregnancies complicated by FGR, there is enhanced activation of maternal peripheral leukocytes when compared to normal pregnancies but lower activation when compared to those complicated by preeclampsia [44]. These differences may explain the fact that patients with preeclampsia have a systemic maternal disease, while those with FGR have a mild activation of the inflammatory system but not a clinically evident disease.

### What is the connection between systemic maternal inflammation and elevated cf-DNA?

The clinical syndrome of preeclampsia is, in part, a consequence of a wide systemic inflammatory response, including endothelium dysfunction [45, 71, 73, 74]. This exaggerated inflammatory response is characterized, among others, by increased expression of pro-inflammatory surface antigens (such as CD11b), increased basal iROS concentration, oxidative burst, and production and release of Neutrophil Extracellular Traps (NETs) [77, 78].

Brinkmann, et al. [79] reported that upon activation by bacterial endotoxin, inflammatory cytokines, or pharmacological agents, neutrophils release granule proteins and chromatin that, together, form extracellular fibers. These NETs degrade virulence factors and kill bacteria. A study in 2006 by Gupta, et al [80] demonstrated that NETs may also be elicited by placental syncytiotrophoblast micro-particles and the intervillous space in placentas of patients with preeclampsia was infiltrated by numerous NETs. Recently, a group from Basel, demonstrated that the increased total cf-DNA in the serum of women with preeclampsia results from increase NETs production by their neutrophils [14]. These findings support our hypothesis that the source of elevated concentrations of total cf-DNA in the serum of women with preeclampsia is mainly derived from the production of NETs resulting from systemic maternal inflammation. The contribution of the placental/fetal unit to the total cf-DNA is probably minor. Thus, we suggest that increased total cf-DNA observed in patients with preeclampsia results from neutrophil activation with the release of NETs full of DNA.

The strength of our study is that we measured the total free cell DNA by using our simple "mix and measure" fluorometric method. The estimated cost of reagents for our assay is 5 cents per sample, much lower than quantitative PCR (QPCR) based assays. In contrast to PCR-based assay that requires DNA extraction from large volume of blood, our assay is performed directly on small volume of serum (0.02 ml). Different from our assay, QPCR assays measure the number of gene copies under investigation and not DNA concentration. Thus, QPCR is affected by extraction losses, by DNA fragmentation, and PCR efficiency. In our view, our simple technique allows for rapid and effective evaluation of patients. We were able to demonstrate similar results as those previously published by more conservative and expensive methods in spite of the small sample size of patients. This assay can be simply applied directly to the relevant biological fluid serum.

## Conclusions

In conclusion, preeclampsia is associated with an increase concentrations of total cf-DNA in the serum compared to normal pregnancies and pregnancies complicated with FGR. The lack of association between maternal serum total cf-DNA concentrations and FGR due to placental origin suggest that the main contribution to the elevated circulation total cf-DNA concentration observed in women with preeclampsia is derived from the maternal compartment and represents the systemic inflammation observed in this syndrome.

## Supporting information

S1 File Clinical information and cell-free DNA levels of the study participants. (XLS)
